# Resolving evolutionary relationships in lichen-forming fungi using diverse phylogenomic datasets and analytical approaches

**DOI:** 10.1038/srep22262

**Published:** 2016-02-26

**Authors:** Steven D. Leavitt, Felix Grewe, Todd Widhelm, Lucia Muggia, Brian Wray, H. Thorsten Lumbsch

**Affiliations:** 1 Integrative Research Center, The Field Museum, 1400 S Lake Shore Drive, Chicago, IL 60605, USA; 2 University of Illinois at Chicago, Department of Biological Sciences, 900 West Taylor St. #1016, M/C 066, Chicago, IL 60612, USA; 3 University of Trieste, Department of Life Sciences, via Giorgieri 10, 34127-Trieste, Italy

## Abstract

Evolutionary histories are now being inferred from unprecedented, genome-scale datasets for a broad range of organismal groups. While phylogenomic data has helped in resolving a number of difficult, long-standing questions, constructing appropriate datasets from genomes is not straightforward, particularly in non-model groups. Here we explore the utility of phylogenomic data to infer robust phylogenies for a lineage of closely related lichen-forming fungal species. We assembled multiple, distinct nuclear phylogenomic datasets, ranging from ca. 25 Kb to 16.8 Mb and inferred topologies using both concatenated gene tree approaches and species tree methods based on the multispecies coalescent model. In spite of evidence for rampant incongruence among individual loci, these genome-scale datasets provide a consistent, well-supported phylogenetic hypothesis using both concatenation and multispecies coalescent approaches (ASTRAL-II and SVDquartets). However, the popular full hierarchical coalescent approach implemented in *BEAST provided inconsistent inferences, both in terms of nodal support and topology, with smaller subsets of the phylogenomic data. While comparable, well-supported topologies can be accurately inferred with only a small fraction of the overall genome, consistent results across a variety of datasets and methodological approaches provide reassurance that phylogenomic data can effectively be used to provide robust phylogenies for closely related lichen-forming fungal lineages.

Novel approaches for obtaining DNA sequence data from across species’ genomes provide unprecedented amounts of information for inferring evolutionary relationships^1^,^2^,^3^,^4^,^5^. Ongoing methodological and analytical advancements facilitate a wide variety of options for generating phylogenomic datasets^6^. However, in most eukaryotic organisms only a small portion of the genome is commonly sampled because of the large size and complexity of their genomes^7^. As our ability to generate genome-scale datasets increases, researchers are required to carefully consider the scale and type of phylogenomic data to address specific research aims.

With the increasing prevalence of large, phylogenomic datasets, incongruence among individual loci appears to be commonplace^8^,^9^. Phylogenetic reconstructions of multi-locus datasets using concatenation may converge on an incorrect topology with strong statistical support^10^,^11^. As researchers move from multi-locus to phylogenomic datasets, the impact of incongruence among individual loci may become more pronounced^8^. Therefore, constructing appropriate datasets and implementing efficient, accurate, and consistent analytical approaches is central to inferring robust hypotheses of evolutionary relationships using genomic data^12^.

In contrast to most other eukaryotes, fungi have relatively small, simple genomes and therefore provide an excellent model for assessing diversification using more comprehensive genomic datasets^13^. While some fungal lineages have played important roles in phylogenomic research^9^,^14^,^15^, lichen-forming ascomycetes have been conspicuously absent. It is estimated that 46% of all ascomycetes form lichen associations^16^, and the accurate inference of evolutionary histories among these symbiotic fungi is of key importance to understanding processes that underlie diversification in this group.

As with most other organismal groups, DNA sequence data has revolutionized our understanding of evolution and diversity in lichen-forming fungi^17^,^18^. However, over the past 20 years most studies of evolutionary relationships have been based on a very limited number of loci and a general reliance on concatenation-based approaches for inferring evolutionary relationships. Furthermore, traditional, phenotype-based approaches for circumscribing species commonly fail to accurately characterize species-level diversity in lichen-forming fungi^17^. Delimiting species-level lineages and inferring relationships among them using molecular sequence data is now a central focus of contemporary research of lichenized fungi.

In order to evaluate the power of phylogenomic data for resolving relationships in problematic species groups of lichen-forming fungi, we investigated the *Rhizoplaca melanophthalma* species complex^19^,^20^,^21^. This group includes at total of eight described species, *R. idahoensis* Rosentr., *R. haydenii* (Tuck.) W.A. Weber, *R. melanophthalma* (DC.) Leuckert, *R. novomexicana* (H. Magn.) Leavitt, Zhao Xin & Lumbsch, *R. parilis* Leavitt *et al.*, *R. polymorpha* Leavitt *et al.*, *R. porteri* Leavitt *et al.*, and *R. shushanii* Leavitt *et al.* While two of the species in the group – *R. melanophthalma sensu stricto* (s. str.) and *R. parilis* – occur across broad, intercontinental distributions, the other species are found almost exclusively in western North America^20^. High levels of intraspecific phenotypic variation is common in this complex, and some species can only be consistently identified using molecular sequence data^19^. In spite of multiple studies implementing multi-locus sequence data, relationships among many lineages within this group remain unresolved^20^,^21^, and questions remain as to the distinction of some of the most recently described species.

The majority of species in the *R. melanophthalma* group diversified during the Pliocene and Pleistocene^20^. Reconstructing evolutionary relationships in groups with recent diversification histories can be confounded by incomplete lineage shorting (ILS) and other evolutionary factors that lead to gene-tree/species-tree incongruence^22^. Previous studies of the *R. melanophthalma* group revealed apparent ILS for a number of taxa in this complex and a general pattern of incongruence among individual gene trees, potentially due to their recent diversification history^20^,^21^.

In this study, we were interested in evaluating the performance of phylogenomic datasets at a variety of scales and using multiple analytical approaches, including concatenation and multispecies coalescent-based species tree methods, to infer evolutionary relationships in lichen-forming fungi. Specifically, we aimed to ascertain if a robust evolutionary hypothesis of relationships in the *Rhizoplaca melanophthalma* group could be inferred using phylogenomic data. Additionally, we used phylogenomic data to evaluate support for species recently described within this group. Our study highlights the promise of phylogenomic data for inferring robust phylogenies for lichen-forming fungi with recent diversification histories.

## Results

### Genomic data, reference genome, and genome assembly

A total of 49.5 Gb of filtered PE reads were generated for this study, and the number of reads for each specimen is reported in Supplementary Table S1. The reference genome assembly from an axenic culture of *R. melanophthalma* (‘mela_REF’) spanned 1070 contigs >5 kilobases (Kb) (longest contig = 363,053 bp), with a N50 size of 46583 and L50 count of 190. These contigs comprised a total of 31.6 of the estimated 38.68 megabase pair (Mb) genome. A CEGMA analysis of the 1070 contigs >5 Kb recovered 93.95% of the complete core eukaryotic genes (CEG) and 96.77% when including both complete and partial CEGs.

### Phylogenomic datasets

**Figure 1:**
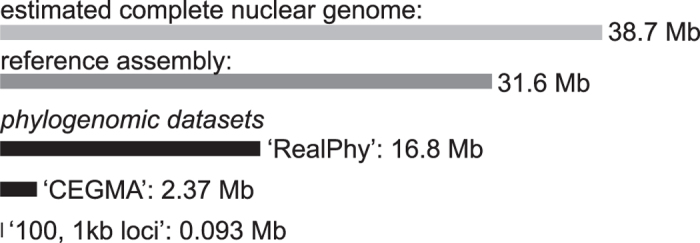
Graphical summary of the three nuclear phylogenomic datasets used to infer relationships in the *Rhizoplaca melanophthalma* species group. The relative sizes of the nuclear datasets are shown in comparison to the estimated size of the complete *R. melanophthalma* reference genome (specimen ‘mela_REF’) and the reference assembly. The ‘RealPhy’ dataset was constructed by mapping reads from all sampled specimens to the *R. melanophthalma* reference assembly using RealPhy v1.12^4^. The ‘CEGMA’ matrix included 430 core eukaryotic genes and associated introns, each with an average length of ca. 5500 base pairs/CEG. The ‘100, 1 Kb loci’ dataset was comprised of 100, 1 kilobase loci selected from each of the 100 largest contigs in the *R. melanophthalma* reference genome assembly.

The ‘RealPhy’, ‘CEGMA’ and ‘100, 1 Kb’ datasets are summarized in Fig. 1, and all data has been deposited to FigShare: (https://dx.doi.org/10.6084/m9.figshare.2120026.v1). The ‘RealPhy’ dataset was comprised of 33 individuals, including *Protoparmeliopsis peltata* and *Rhizoplaca subdiscrepans*, and comprised 16.8 Mb, representing 53% of the reference genome (contigs >5 Kb). An average of 70.8% of the reference genome was covered by each specimens within the *R. melanophthalma* complex in the RealPhy assembly, although the proportion of genome coverage was much lower for *P. peltata* and *R. subdiscrepans*, at 4.7% and 18.6%, respectively (Supplementary Table S2). For specimens from the *R. melanophthalma* species complex, the proportion of genome coverage relative to the reference ranged from 51.2% for the specimen ‘poly_8807-3’ (*R. polymorpha*) to 91.7% for ‘mela_8801’ (*R. melanophthalma*); and 99.99% for reads from the axenic culture mapped back to the reference. The ‘CEGMA’ matrix included 430 core eukaryotic gene (CEG) regions, including introns and small portions of upstream and downstream regions, with an average length of ca. 5500 bp/CEG region, for a total size of 2.37 Mb. A total of 303 CEGs (exons only) passed filtering requirements and were also analyzed (Supplementary text online). The ‘100, 1 Kb loci’ dataset comprised 92.67 Kb, after excluding sites with unsuccessful mappings or insufficient coverage. Phylogenetic informativeness (PI) of the coding regions from the 303 CEGs and loci from the ‘100, 1 Kb’ dataset are shown in Supplementary Fig. S1.

### Concatenated phylogenomic inferences

**Figure 2:**
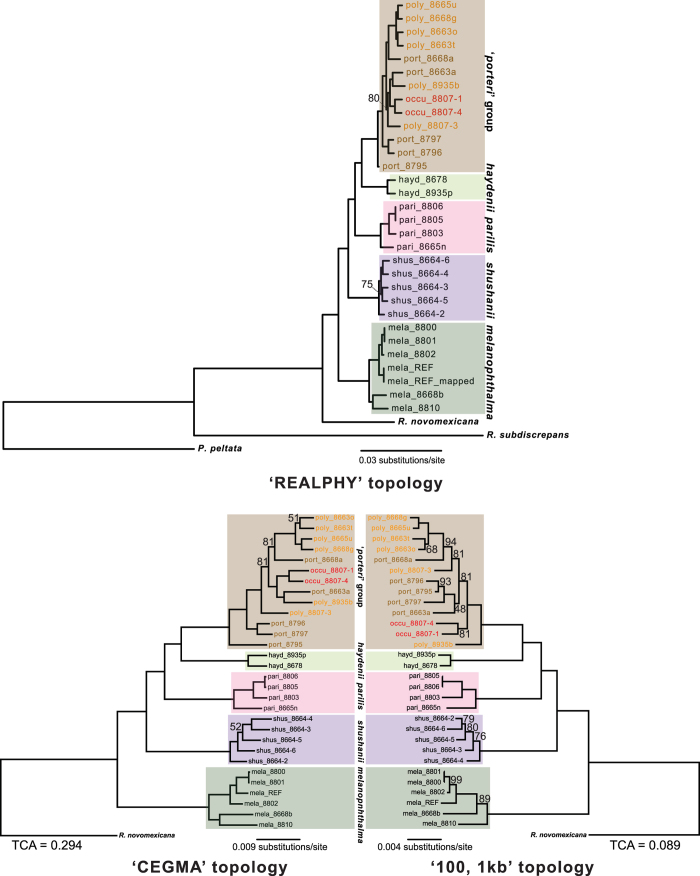
Topologies inferred from the three nuclear phylogenomic datasets. The top panel shows the topology from the ‘RealPhy’ dataset comprised of a 16.8 Mb alignment. The bottom panel shows a comparison between topologies inferred from the ‘CEGMA’ (2.37 Mb) and ‘100, 1 Kb loci’ (0.93 Mb). Specimens representing each species are highlighted with a corresponding color for comparison. The ‘*porteri* group’ comprised of specimens representing *R. occulta* (‘occu’), *R. polymorpha* (‘poly’), and *R. porteri* (‘port’) is highlighted in brown; and the color of the specimens label corresponds to each of the three distinct taxa. Bootstrap values for each node equaled 100%, unless otherwise noted. Relative tree certainty, including all conflicting partitions (TCA), estimated from individual gene trees is reported for the ‘CEGMA’ and ‘100, 1 Kb loci’ datasets.

Phylogenies inferred from concatenated nuclear phylogenomic datasets revealed highly similar relationships (Fig. 2). Species were recovered as monophyletic clades with 100% BS support in all topologies, with the exception of the monophyletic, well-supported ‘*porteri* group’, which was comprised of *R. occulta*, *R. polymorpha*, and *R. porteri*. There was relatively little divergence among specimens recovered within this group; and species were not recovered as monophyletic, with the exception of *R. occulta* (Fig. 2). The degree of incongruence between individual gene trees across the entire phylogeny was estimated using the tree certainty score (TCA). TCA values describe the global degree of incongruence between individual gene trees in the set. We report the relative values normalized by the maximum TCA value for a given phylogeny, ranging from 0.0 (complete incongruence between all individual gene trees) to 1.0 (complete congruence between all gene topologies). The TCA score was 0.294 for the ‘CEGMA’ topology and 0.089 for the ‘100, 1 Kb’ topology. A partitioned ML analysis of coding regions from the ‘CEGMA’ dataset (intron excluded) and a ML analysis of the third codon position resulted in topologies that were highly similar to other topologies (Supplementary Fig. S2).

**Figure 3:**
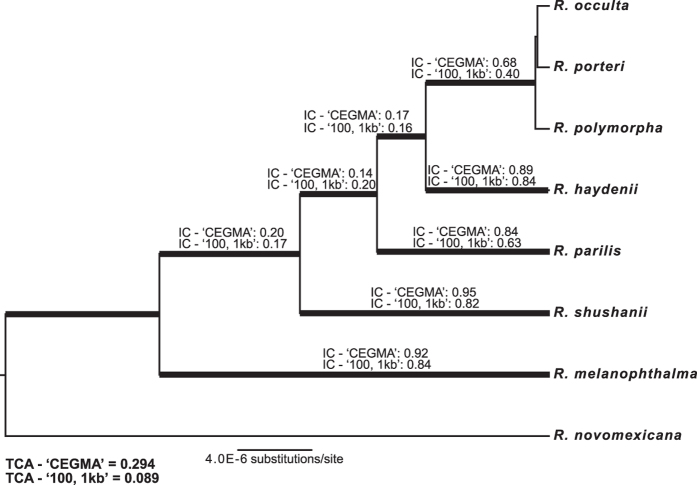
Species tree for the *Rhizoplaca melanophthalma* species group inferred from a dataset comprised of loci ‘51’–‘100’ from the ‘100, 1 Kb’ dataset using the program *BEAST. Species tree analyses of the ‘CEGMA’ and ‘100, 1 Kb’ datasets using ASTRAL-II and SVDquartets + PAUP* resulted in identical topologies with 100% support, with the exception of relationships among closely related taxa within the ‘*porterii* clade’. Internode certainty (IC) on each branch and the relative tree certainty (TCA), estimated from individual gene trees, are reported from ML analyses of ‘CEGMA’ and ‘100, 1 Kb loci’ datasets. The IC value of a given internode reflects its specific degree of incongruence; and the TCA values characterize the global degree of incongruence between trees.

The degree of incongruence for each internode in a set of gene trees was quantitatively characterized using internode certainty (IC) values (Fig. 3). The IC score calculates the degree of certainty for a given internode by considering the frequency of the bipartition defined by the internode in a given set of trees jointly with that of the most prevalent conflicting bipartition in the same tree. IC values at or near 1 indicate the absence of any conflict at the internode; whereas IC values at or near 0 indicate that one or more conflicting bipartition have almost equal support. While *R. melanophthalma* s. str., *R. shushanii*, *R. parilis*, *R. haydenii*, and the ‘*porteri* group’ were recovered as monophyletic in the majority of individual CEG topologies and topologies inferred from individual loci from the ‘100, 1 Kb loci’ dataset, high levels of incongruence among individual gene topologies were observed for relationships among these clades (summarized in Fig. 3).

### Phylogenomic species tree inferences and speciation probabilities

**Figure 4:**
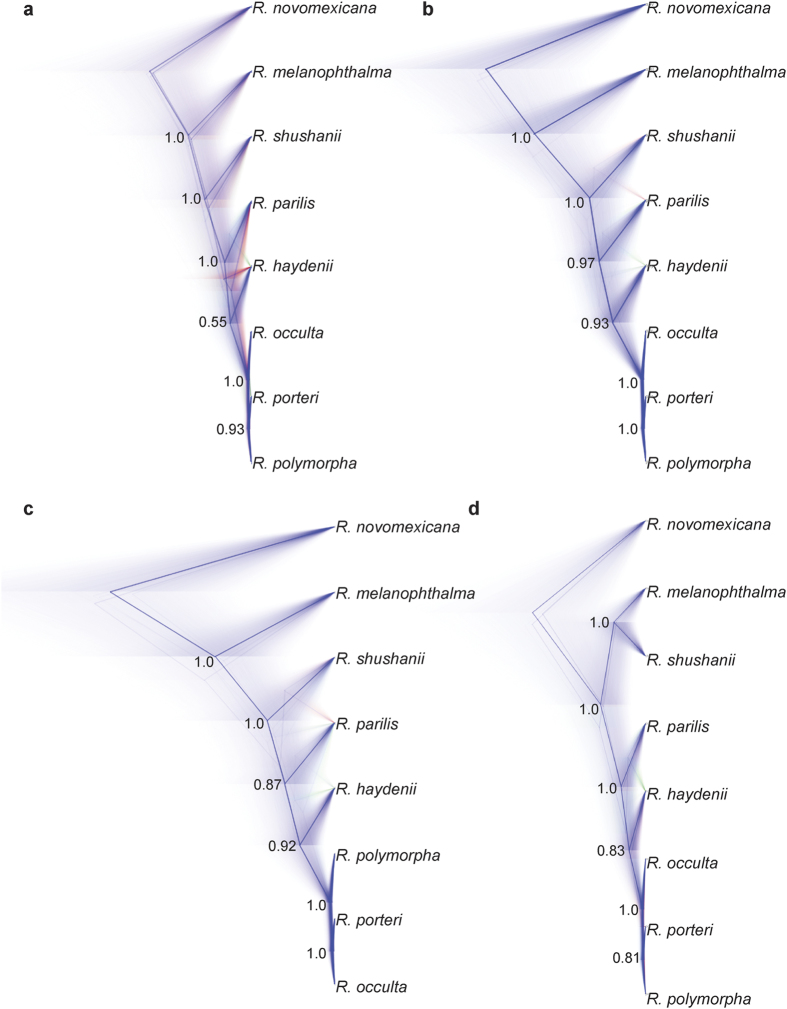
*BEAST analyses of the four, 25-locus subsets from the ‘100, 1 Kb loci’ dataset. (**a**) species tree inferred from loci ‘1’–‘25’; (**b**) species tree inferred from loci ‘26’–‘50’; (**c**) species tree inferred from loci ‘51’–‘75’; and (**d**) species tree inferred from loci ‘76’–‘100’. A consensus topology (calculated as the average of the branch length for all trees with the same topology) is superimposed on a cloudogram of the entire posterior distribution of species trees (after burn-in) for each *BEAST analysis. The most popular branching pattern is shown in blue, the next most popular is shown in red, and the third most popular in green. Posterior probabilities are included for each node.

Species tree analyses of the ‘CEGMA’ and ‘100, 1 Kb’ datasets using the summary coalescent-based species tree inference methods ASTRAL-II and SVDquartets + PAUP* resulted in identical topologies with 100% support, with the exception of relationships among closely related taxa within the ‘*porterii* group’ (Fig. 3). The SVDquartets + PAUP* analyses of the two 50-locus datasets derived from the ‘100, 1 Kb loci’ dataset resulted in identical topologies and identical nodal support values (data not shown). Branching patterns among *R. melanophthalma* s. str., *R. shushanii*, *R. parilis*, *R. haydenii*, and the ‘*porteri* group’ were identical to those inferred using ASTRAL-II and SVDquartets, but strong nodal support for all nodes was only observed in the dataset comprised of loci ‘51’–‘100’ (Supplementary Fig. S3). Therefore, we used the *BEAST topologies from the dataset comprised of loci ‘51’–‘100’ to represent the species tree, including branch lengths, for the *R. melanophthalma* group (Fig. 3). However, low effective sample size (ESS) values were observed for most parameters in *BEAST analyses of the 50-locus datasets, although ESS values for likelihood were above 200. ESS values were generally >150 for most parameters in each of the *BEAST analyses of the 25-locus subsets. Here we report the topologies from the *BEAST analyses of the four 25-locus subsets (Fig. 4). In three of the four cases, branching patterns among *R. melanophthalma* s. str., *R. shushanii*, *R. parilis*, *R. haydenii*, and the ‘*porteri* group’ were identical to those inferred from the summary coalescent methods ASTRAL-II and SVDquartets + PAUP* and concatenated analyses of the ‘RealPhy’, ‘CEGMA’, and ‘100, 1 Kb loci’ datasets. However, support values were less than 0.95 at a number of nodes, and the topology inferred from one of the four 25-locus subsets differed from the other three with strong statistical support (Fig. 4d). Furthermore, different relationships among species in the ‘*porteri* group’ were recovered with strong support in the *BEAST analyses of the 25-locus subsets.

### Bayesian species validation

BP&P analyses conducted on the ‘100, 1 Kb’ dataset and two subsets of 50 loci resulted in speciation probabilities equaling 1.0 for all species, including strong support for the distinction of closely related species in the ‘*porteri* group’ – *R. occulta*, *R. polymorpha*, and *R. porteri*.

## Discussion

Phylogenomic datasets provide unprecedented potential for reconstructing evolutionary histories. However, identifying the appropriate scale and loci for sampling genomes is uncertain in most empirical studies. In this study, we show that consistent, well-supported topologies were reconstructed from distinct phylogenomic datasets, ranging from less than 100 Kb to nearly 17 Mb (Fig. 1), for a lineage of lichen-forming ascomycetes comprised of closely related species. In spite of high levels of incongruence among genomic regions, a robust hypothesis of evolutionary relationships was inferred for the *Rhizoplaca melanophthalma* group using both concatenation (Fig. 2) and coalescent-based species tree methods (Fig. 3). Consistent results across a variety of datasets and methodological approaches provide reassurance that phylogenomic data can effectively be used to provide robust phylogenies for lichen-forming fungal lineages even in cases of rampant incongruence among genomic regions.

The general impact of missing data in phylogenomic datasets is not well characterized. One potential limitation to approaches that utilize mapping short reads to a reference genome, such as those implemented in this study, is the fact that the proportion of successfully mapped reads to a reference decreases with increasing divergence. For example, in this study we were unable to generate data for the ‘CEGMA’ datasets from the two outgroup taxa (*Protoparmeliopsis peltata* and *Rhizoplaca subdiscrepans* using the ‘map_n_extract’ pipeline (https://github.com/felixgrewe/map_n_extract/) due to largely unsuccessful read mapping for these. Similarly, the proportion of the reference genome that was successfully mapped using reads from *P. peltata* and *R. subdiscrepans* was quite low in comparison to the ingroup taxa (Supplementary Table S2). However, overall we attempted to limit the amount of missing data to reasonable levels. In the RealPhy assembly, we limited the amount of missing data per column to 20%; and the overall amount of missing data was well below this threshold. Similarly, the complete ‘CEGMA’ dataset comprised of 430 CEGs (and associated introns) included ca. 15.5% missing data, although the reduced 303 CEGs (exons only) that passed filtering requirements was comprised of only 0.92% missing data. The ‘100, 1 Kb’ dataset included less than 10% missing data, and only 6% missing data when considering the ingroup alone. Studies implementing approaches similar to that which we propose here will be limited to clades with relatively recent diversification histories.

While researchers are now able to generate large, relatively comprehensive phylogenomic data matrices for fungal lineages^15^, we show that consistent, well-supported topologies can be accurately inferred with only a small fraction of the overall genome in the closely related *R. melanophthalma* group (Fig. 1). Furthermore, the use of smaller, better-characterized phylogenomic datasets (e.g., ‘CEGMA’ and ‘100, 1 Kb’) facilitates inference under the multi-species coalescent model with a number of contemporarily available approaches^23^,^24^. Coalescent-based species tree methods are theoretically preferable to concatenated gene tree approaches^10^. However, some coalescent-based species tree methods may be impractical for large phylogenomic datasets^25^.

The hierarchical coalescent model implemented in *BEAST is generally not considered to be appropriate for genome-scale analyses due to computation constraints required to implement this full-coalescent model^26^. Therefore, summary methods, such as ASTRAL-II^24^ and SVDquartets^23^, provide a computationally efficient approach that accounting for incomplete lineage sorting (ILS) under the multi-species coalescent model and have been shown to be statistically consistent^24^,^27^. While the accuracy of summary methods increases with the number of sampled loci^12^, *BEAST has been shown to provide consistent, accurate topologies using far fewer loci^28^,^29^. However, here we demonstrate that small phylogenomic datasets (e.g., 25 loci comprising ca. 23 Kb nucleotide position characters) provided inconsistent inferences, both in terms of nodal support and topology (Fig. 4). Furthermore, *BEAST analyses of larger, 50-locus datasets showed evidence of poor mixing and resulted in low ESS values, although topologies and nodal support values were consistent with the inference from ASTRAL-II and SVDquartets + PAUP* (data not shown).

In contrast to the full, hierarchical multi-species coalescent approach implemented in *BEAST^30^, the summary coalescent methods ASTRAL-II and SVDquartets + PAUP* provide computationally efficient approaches to accurately infer species trees in the presence of ILS^12^. In our empirical study of the *R. melanophthalma* species group, both methods provided identical, well-support topologies (Fig. 3). One advantage of SVDquartets + PAUP*, relative to ASTRAL-II, is that is does not require the intermediate step of inferring individual gene topologies and analyses of phylogenomic datasets can be performed with minimal computational time. However, ASTRAL-II has been shown to outperform SVDquartets in cases with increasing ILS^12^. Alternatively, when levels of ILS are low, concatenation can perform as well or better than coalescent-based species tree methods^12^,^27^,^31^.

In our study of the *Rhizoplaca melanophthalma* group, loci in the ‘CEGMA’ and ‘100, 1 Kb’ datasets were arbitrarily chosen without optimization for selecting the most informative loci. Although our phylogenomic datasets provided consistent inferences of evolutionary relationships (Fig. 2), identifying and selecting a narrow range of specific, optimal loci for reconstructing phylogenies may improve scalability, both in terms of the number of specimens that can be sampled and the evolutionary breadth of sampled taxa^1^,^8^,^32^. Exploratory phylogenetic analyses of ten loci with the highest PI corresponding to the timing of relatively recent diversification events in the *Rhizoplaca melanophthalma* group (0.003–0.009 in the Supplementary Fig. S1) provided widely inconsistent topologies relative to those inferred from more comprehensive phylogenomic datasets (results not shown). In contrast, we found that phylogenies inferred from ten loci with the lowest PI were largely consistent with topologies reconstructed from phylogenomic datasets (results not shown). The unexpected results from these exploratory analyses highlight the fact that additional research will be required to determine effective approaches for selecting the most appropriate loci for multilocus phylogenetic reconstructions from phylogenomic data.

Phylogenetic accuracy using non-specific data can be considerably influenced by the size of data and choice of tree inference methods^33^. Selecting question-specific genes and loci that contain a minimal amount of non-phylogenetic signal can substantially reduce incongruence^8^. Bootstrapping analyses will commonly provide very high levels of nodal support, even in cases of rampant conflict/incongruence among individual loci^9^. Therefore, reporting quantitative metrics of incongruence among loci in phylogenomic datasets, in addition to traditional nodal support assessments, provides a more comprehensive perspective of how the data support a specific phylogenetic hypothesis^34^.

A number of recent studies have revealed largely undifferentiated genomes among some currently recognized species, particularly in cases of recent radiations^35^. In this study, the majority of species in the *R. melanophthalma* group were recovered as monophyletic clades (*R. novomexicana*, *R. haydenii*, *R. melanophthalma*, *R. parilis*, *R. shushanii*), while *R. occulta*, *R. polymorpha*, and *R. porteri* appear to belong to a very recent, rapid radiation and were not recovered as monophyletic (Figs. 2 and 3). Strikingly, these taxa can only be effectively discriminated using molecular sequence data from the ribosomal cistron^19^. These species were diagnosed with high speciation probabilities in previous studies, and *R. porteri* can also be discriminated from species in the *R. melanophthalma* group by the absence of a group I intron at the 3′ end of the 18S rDNA^20^,^21^. Specimens within this group are easily identifiable using internal transcribed spacer region (ITS), the official barcoding marker for fungi^36^. Here, the BP&P^37^ analyses of the ‘100, 1 Kb’ data provided unambiguous support for the validity of these taxa as distinct, species-level lineages, in spite of the fact that they were not recovered as monophyletic in any phylogenetic analysis. BP&P has shown to perform accurately across a broad range of scenarios^38^,^39^. In this study, the 100 independent nuclear loci analysed using BP&P provide unprecedented amounts of data supporting the distinction of these species. This data suggests that rDNA may effectively track recent, rapid radiations that are otherwise not reflected in phylogenomic datasets. Alternatively, the ‘*porteri* group’ may correspond to single single-level lineage and not three separate taxa. Distinct patterns in rDNA may reflect stochastic evolutionary events, rather than tracking recent divergence events among lineages. Error rates in BP&P may be high when individuals are incorrectly assigned to populations^40^, and specimen identification of *R. occulta*, *R. polymorpha*, and *R. porteri* is based on rDNA sequence data. Future studies characterizing rDNA evolution and intragenomic variation in the *R. melanophthalma* group, coupled with identifying potential genes/genomic regions associated with divergence among these taxa, will be required ascertain if *R. occulta*, *R. polymorpha*, and *R. porteri* do, in fact, represent distinct species.

In conclusion, our empirical study of a group of closely related lichen-forming fungal species demonstrates the utility of genome-scale datasets for inferring robust hypotheses of evolutionary relationships. We provide additional evidence that single-copy CEGs derived from the eukaryotic orthologous groups (KOGs) provide a valuable set of markers for phylogenomic markers^41^. In contrast to other markers that are commonly used in phylogenomic research – e.g., RADseq loci^7^, ultra conserved elements^1^, etc. – CEGs provide a highly reliable set of well-characterized gene regions that can consistently be applied across eukaryotes. We provide a standardized pipeline for extracting CEGs to facilitate their use in phylogenomic research (https://github.com/felixgrewe/map_n_extract/).

## Materials and Methods

A complete description of materials and methods can be found as Supplementary Information (Supplementary text S1), and below we summarize our methodological approach.

### Culture Isolation for reference genome

For this study, an axenic culture representing the mycobiont taxon *R. melanophthalma* s. str. was used to provide reference genomic data. The mycobiont was cultured from a single areole following the ‘thallus fragments’ method^42^. The cultured strains are deposited at the University of Graz in the culture collection of the author LM (LMCC) and are preserved both as fresh cultures and as cryostocks.

### Taxonomic sampling

A total of 30 specimens representing eight of the nine described species within the *Rhizoplaca melanophthalma* species complex were collected from sites throughout western North America (Supplementary Table S1; Supplementary Fig. S4). We were unable to obtain fresh material representing the vagrant taxon *R. idahoensis*, which has previously been shown to be closely related to *R. haydenii*^21^. Two additional *Rhizoplaca* s. lat. species were included as outgroups – *Protoparmeliopsis peltata* (Ramond) Arup, Zhao Xin & Lumbsch, and *R. subdiscrepans* (Nyl.) R. Sant. – to infer the outgroup for the *R. melanophthalma* complex. Based on the topology inferred from the ‘RealPhy’ dataset, *R. novomexicana* was used as the outgroup for the *R. melanophthalma* complex.

### DNA extraction and sequencing

DNA was extracted using either a CTAB protocol^43^ or the Prepease DNA Isolation Kit (USB, Cleveland, Ohio, USA). The identity of each DNA extraction was confirmed by sequencing the nuclear ITS rDNA region using the primers ITS1f^44^ and ITS4^45^. Genomic libraries for the *R. melanophthalma* s. str. reference culture, *R. novomexicana*, *R. subdiscrepans*, and *P. peltata* were prepared using Illumina’s TruSeq DNA LT Sample Prep Kit, following the manufacture’s instructions for 250-bp paired-end (PE) reads with a 550-bp insert size. Libraries were sequenced on Illumina’s MiSeq platform using the Illumina’s MiSeq v2 Reagent Kit at the Pritzker Laboratory for Molecular Systematics at the Field Museum (Chicago, IL, USA). Library preparation and sequencing of genomic DNA from the remaining 30 samples was completed at the Georgia Genomics Facility (http://dna.uga.edu/). Libraries were constructed using an in-house method and libraries were pooled and sequenced on a single lane of Illumina HiSeq2000, 100-bp paired-end reads with a 350-bp insert size.

### Read filtering and genome assemblies

All PE reads were filtered using TRIMMOMATIC v0.33^46^ before assembly to remove low quality reads and/or included contamination from Illumina adaptors. The genome size of *R. melalnophthalma* s. str. was estimated from filtered PE reads using the perl script “estimate_genome_size.pl” (https://github.com/josephryan/estimate_genome_size.pl).

A reference draft genome was assembled using PE Illumina reads from the axenic *R. melanophthalma* s. str. culture using the RAY v2.3.1 assembler^47^,^48^ with a kmer value of 41 and the remaining parameters set to default values. An exploratory comparison of assemblies using the RAY and SPAdes v3.1.1^49^ assemblers, implementing a variety of kmer values, indicated that the selected RAY assembly was most complete in terms of core eukaryotic genes (CEG)^50^ and closest to the estimated genome size.

### Phylogenomic data matrices

Three phylogenomic datasets were assembled for this study: (i) ‘nuRealPhy’; ‘CEGMA’; and ‘100, 1 Kb’ (Fig. 1). The most comprehensive nuclear phylogenomic dataset – ‘RealPhy’ – was constructed using the program RealPhy v1.12^4^. Excluding non polymorphic sites has been shown to potentially bias phylogenetic inference^4^. RealPhy addresses this problem by including invariant sites in reconstructed multiple sequence alignments and can combine alignments from mappings to multiple reference sequences to further minimize potential biases^4^. After excluding the contig containing the mitochondrial genome, all contigs from the reference (‘mela_REF’) genome assembly larger than five Kb were used as the reference. PE reads from all the remaining specimens were mapped to the reference using the following parameters in RealPhy, implementing Bowtie 2.1.0 for read mapping and the following parameters: -readLength 75 –perBaseCov 5 –gapThreshold 0.2 with the remaining parameters set to default values. With the –gapThreshold parameter set to 0.2, each site had no more than 20% missing data.

The ‘CEGMA’ genomic data matrix was constructed using the Core Eukaryotic Gene Mapping Approach (CEGMA;^50^,^51^). Proteins included in CEGMA represent 458 eukaryotic orthologous groups (KOGs) that are conserved among eukaryotes and provide a potentially informative phylogenomic markers^41^. Consensus sequences for each CEG region, including introns and small portions of upstream and downstream regions, were aligned using the program MUSCLE^52^. The ‘CEGMA’ matrix was comprised of concatenated alignments from a total of 430 CEGs. Exon regions from individual CEG consensus sequences passing filtering parameters were also extracted for partitioned phylogenetic analysis. The entire pipeline is available as a GitHub repository (https://github.com/felixgrewe/map_n_extract/).

The ‘100,1 Kb loci’ dataset was assembled from a single 1 Kb genomic region selected from each of the 100 largest contigs from the RAY assembly of the reference genome. Mappings of the *P. peltata* reads to the 100 largest contigs in the reference genome were examined to identify the first 1 Kb regions covered without gaps. Each genomic region was evaluated for uniform coverage across the locus and similar coverage among loci to avoid the selection of paralogous or repetitive genomic regions. PE reads from all specimens were mapped to these 100, 1 Kb markers from the reference genome using RealPhy v1.12^4^. We assessed phylogenetic informativeness (PI) for each locus in the ‘100, 1 Kb loci’ dataset using the PhyDesign web interface^53^.

### Phylogenomic inference

Phylogenetic relationships were inferred using maximum likelihood (ML) and multi-species coalescent species tree approaches. ML phylogenetic relationships were inferred from the complete the ‘RealPhy’, ‘CEGMA’, and ‘100, 1 Kb’ datasets using the program RAxML v8.2.3^54^. Individual ML topologies were also inferred for each individual locus in the ‘RealPhy’ and ‘CEGMA’ datasets. Nodal support was evaluated using 1000 bootstrap pseudo-replicates. We inferred the phylogeny of the ‘100,1 Kb loci’ datasets using partitioned ML analyses in RAxML. We used the program PartitionFinder^55^ to infer the most appropriate partitioning for both ‘100,1 Kb loci’ datasets.

Incongruence among individual gene topologies from the ‘CEGMA’ and ‘100, 1 Kb’ datasets was evaluated using the internode certainty (IC) and relative tree certainty (TCA) metrics^34^. The IC value of a given internode reflects its specific degree of incongruence, and the TCA value characterized the global degree of incongruence between trees. Individual gene trees from the ‘CEGMA’ and ‘100, 1 Kb’ datasets were estimated using RAxML v8.2.3 as described above.

### Species tree inference from phylogenomic data

Because phylogenetic inferences from concatenated data may differ from species tree approaches^56^, we inferred species-trees for the *R. melanophthalma* group using three approaches based on the multispecies coalescent model: the summary coalescent approaches ASTRAL-II^24^ and SVDquartets^23^, along with a Bayesian hierarchical approach, *BEAST^30^.

We used the summary coalescent model ASTRAL-II v4.7.8^24^ to inter a species tree from two sets of unrooted gene trees: (1) gene trees inferred from the alignments of the 430 CEGs (and associated introns) identified in this study; and (2) gene trees inferred from each of the ‘100, 1 Kb’ loci. ASTRAL-II estimates a species tree given a set of unrooted gene trees and has been shown to be statistically consistent under the multi-species coalescent model^24^. Individual ML gene trees and bootstrap replicates were inferred using RAxML v8.2.3. We used ASTRAL-II with multi-locus bootstrapping (MLBS) option.

A second summary method, SVDquartets^23^, infers the quartet trees for all subsets of four species using unlinked multi-locus data, assigning a score to each of the three possible quartet topologies. We ran SVDquartets as implemented in PAUP* v4.0a146 using the ‘CEGMA’ and ‘100, 1 Kb’ datasets, independently. We also ran SVDquartets on two subsets of the ‘100, 1 Kb’ dataset, arbitrarily dividing the 100 loci, into two 50-locus datasets to assess the performance SVDquartets + PAUP* using smaller genomic datasets.

We also estimated species trees using the hierarchical Bayesian model implemented in *BEAST v. 1.8.2^30^. *BEAST estimates a species tree directly from the sequence data, incorporating the coalescent process, uncertainty associated with gene trees, and nucleotide substitution model parameters^30^. *BEAST is computationally intensive, and we arbitrarily divided the ‘100, 1 Kb’ dataset, into four 25-locus datasets in order for the analyses to be computationally feasible. Exploratory analyses of the complete ‘100, 1 Kb’ dataset and two 50-locus datasets derived from the complete matrix failed to converge and were not considered further. Nucleotide substitution models were inferred for each locus using the program PartitionFinder v1.1.1^55^ with Akaike Information Criterion model selection. For all *BEAST analyses we selected the birth-death speciation prior, implementing a relaxed lognormal molecular clock. Two independent MCMC analyses were run for a total of 100 million generations, sampling every 1500 steps, and excluding the first 25% of generations from each run as burn-in. We assessed convergence by examining the likelihood plots through time using Tracer v. 1.6^57^, and the effective sample sizes (ESS) of parameters. Posterior probabilities (PP) of nodes were computed from sampled trees after burn-in.

### Bayesian species validation

We estimated the marginal posterior probability of speciation from the individual loci in the ‘100, 1 Kb’ dataset using the program BP&P v3^37^,^58^. This method accommodates the species phylogeny as well as lineage sorting due to ancestral polymorphism. We used a conservative combination of priors that should favor fewer species by assuming large ancestral population sizes and relatively shallow divergences among species with algorithm 0. Species trees estimated in SVDquartets + PAUP* and *BEAST analysis were used as the fully resolved guide trees, differing only in the placement of taxa within the closely related ‘*porteri* group’ (see Results). Running the rjMCMC analysis for 50,000 generations with a burn-in of 50,000 produced consistent results across independent analyses initiated with different starting seeds and species trees. We also ran BP&P as described above on two subsets of the ‘100, 1 Kb’ dataset, arbitrarily dividing the 100 loci, into two 50-locus datasets to assess if BP&P provided consistent results across different datasets.

## Additional Information

**How to cite this article**: Leavitt, S. D. *et al.* Resolving evolutionary relationships in lichen-forming fungi using diverse phylogenomic datasets and analytical approaches. *Sci. Rep.* **6**, 22262; doi: 10.1038/srep22262 (2016).

## Supplementary Material

Supplementary Information
