# Supplementary material for: Resolving evolutionary relationships in lichen-forming fungi using diverse phylogenomic datasets and analytical approaches
Source: Sci Rep. 2016 Feb 26;6:22262. doi: 10.1038/srep22262 (PMC4768097; doi:10.1038/srep22262)
Supplement: Supplementary Information [file srep22262-s1.doc]

**Resolving evolutionary relationships in lichen-forming fungi using diverse phylogenomic datasets and analytical approaches**

**Supplementary Information**

Steven D. Leavitt^1,*,+^, Felix Grewe^1,+^, Todd Widhelm^1,2^, Lucia Muggia^3^, Brian Wray^1^, and H. Thorsten Lumbsch^1^

^1^Science and Education, The Field Museum, 1400 S Lake Shore Drive, Chicago, IL 60605 USA

^2^University of Illinois at Chicago, Department of Biological Sciences, 900 West Taylor St. #1016, M/C 066, Chicago, IL 60612

^3^University of Trieste, Department of Life Sciences, via Giorgieri 10, 34127-Trieste, Italy

[^*^sleavitt@fieldmuseum.org](mailto:*sleavitt@fieldmuseum.org)

^+^these authors contributed equally to this work

**Supplementary Figure S1**. Graphical overview of the phylogenic informativeness (PI) of 403 individual loci estimated the PhyDesign online tool (http://phydesign.townsend.yale.edu/). Relative time units are shown on the X-axis and profiles of the phylogenetic informativeness per site are shown on the Y-axis. PI of the 100 loci from the ‘100, 1kb’ dataset are shown in green; and PI of the 303 GEGs extracted from the ‘CEGMA’ dataset are shown in grey.

**Supplementary Figure S2**. Topologies inferred from 303 core eukaryotic genes (CEGs) extracted from the ‘CEGMA’ dataset. a) Maximum likelihood (ML) topology inferred from a partitioned RAxML analysis of the 303 CEGs; b) ML topology inferred from the third codon position of the 303 CEGs. All bootstrap support values equaled 100% unless otherwise noted.

**
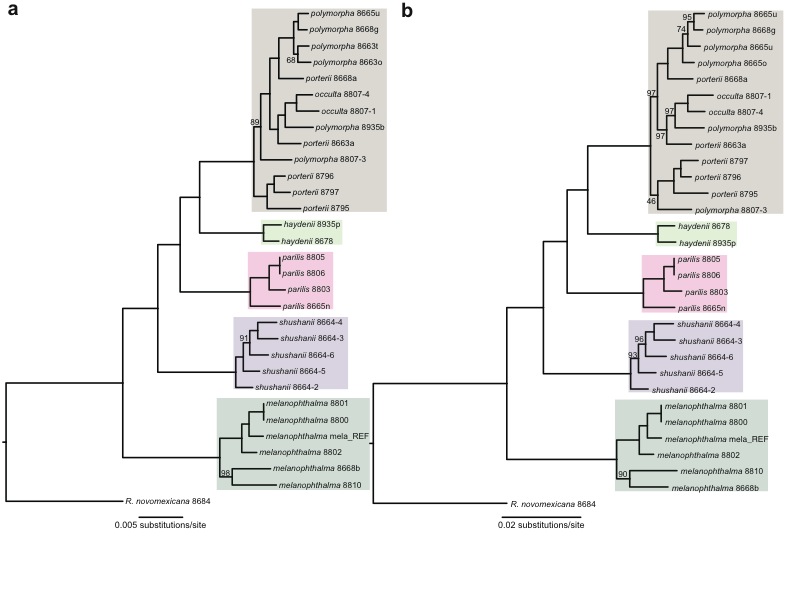
**

**Supplementary Figure S3**. *BEAST analyses of the two, 50-locus subsets from the ‘100, 1kb loci’ dataset. **a** - species tree inferred from loci ‘1’–‘50’; and **b** - species tree inferred from loci ‘51’–‘100’. Consensus topologies (calculated as the average of the branch length for all trees with the same topology) is superimposed on a cloudogram of the entire posterior distribution of species trees after burn-in for each *BEAST analysis. Posterior probabilities are included for each node.

**Supplementary Figure S4**. Geographic distribution of sampled specimens within the *Rhizoplaca melanophthalma* species complex included in the current study. List of sampled taxa: *R. haydenii* (site 1.); *R. melanophthalma* (sites 5, 8, 10, & R); *R. novomexicana* (site 12); *R. parilis* (sites 2, 3 & 6); *R. polymorpha* (sites 1, 4, 5, 6 & 7); *R. porterii* (sites 4, 5 & 9); and *R. shushanii* (site 11). The *R. melanophthalma* reference culture was derived from a specimen collected at site ‘R’. The map was generated using the online mapping tool SimpleMappr (<http://www.simplemappr.net/>).


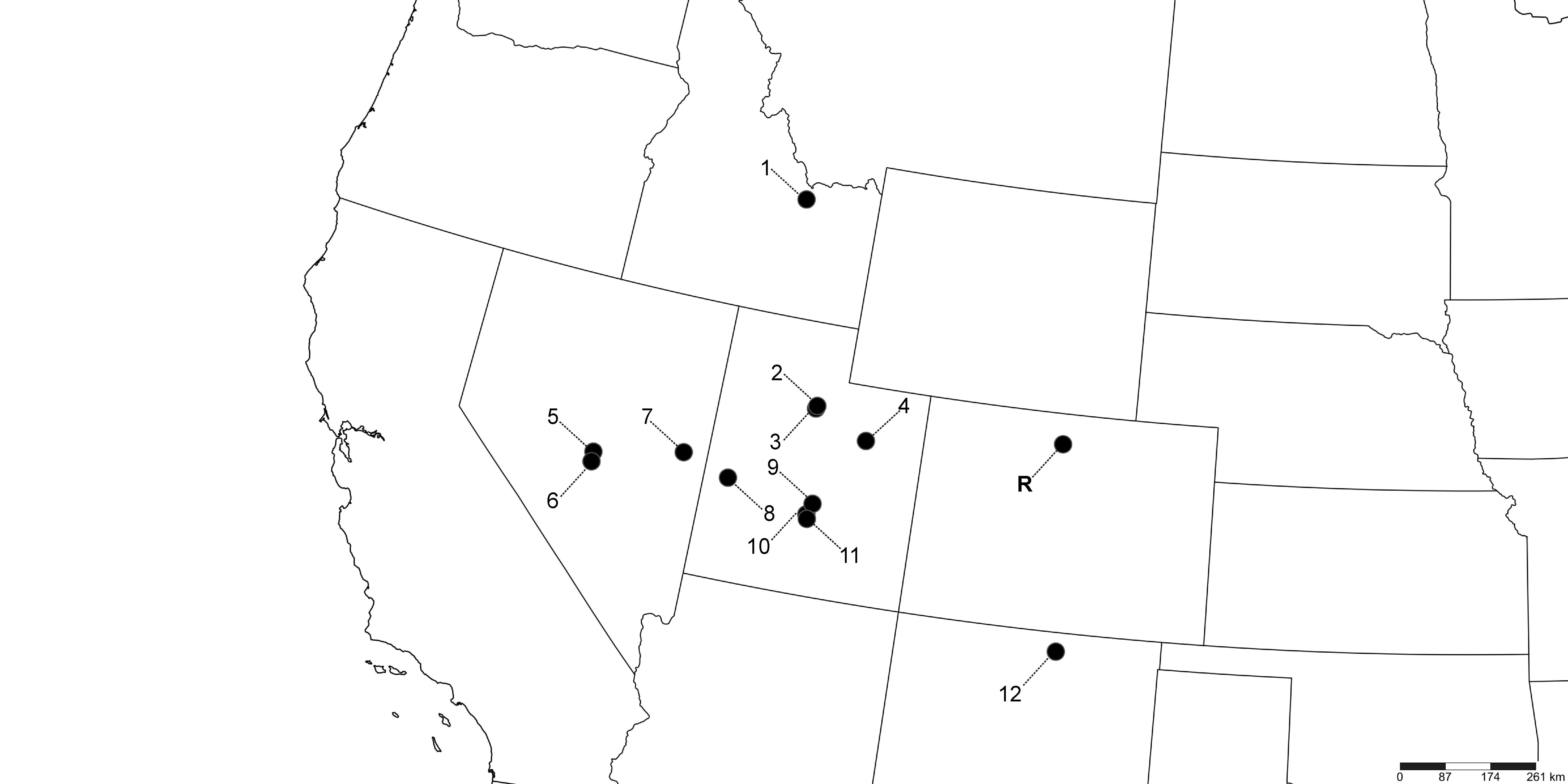


**Supplementary Table S1.** Collection information for each sampled specimen.

| **TAXON** | **DNA_CODE** | **LOCALITY** | **LAT.** | **LON.** | **SITE (Fig. S4)** | **# of filtered reads** |
| --- | --- | --- | --- | --- | --- | --- |
| *R. haydenii* | hayd_8678 | USA, Idaho, Lemhi Valley, vic. of Birch Creek Campgroup | 44.16 | -112.92 | 1 | 10,226,038  (2×100) |
| *R. haydenii* | hayd_8935p | USA, Idaho, Lemhi Valley, vic. of Birch Creek Campgroup | 44.16 | -112.92 | 1 | 8,549,740  (2×100) |
| *R. melanophthalma* | mela_8668b | USA, Nevada, Nye Co.: Humboldt-Toiyabe N.F., Table Mountain Wilderness Area, vic. of Mosquito Creek | 38.81 | -116.68 | 5 | 9,501,798  (2×100) |
| *R. melanophthalma* | mela_8800 | USA, Utah, Wayne Co.: Thousand Lakes Mountain, vic. of Forsyth Reservoir | 38.49 | -111.51 | 10 | 6,211,434  (2×100) |
| *R. melanophthalma* | mela_8801 | USA, Utah, Wayne Co.: Thousand Lakes Mountain, vic. of Forsyth Reservoir | 38.49 | -111.51 | 10 | 12,371,904  (2×100) |
| *R. melanophthalma* | mela_8802 | USA, Utah, Wayne Co.: Thousand Lakes Mountain, vic. of Forsyth Reservoir | 38.49 | -111.51 | 10 | 11,653,316  (2×100) |
| *R. melanophthalma* | mela_8810 | USA, Utah, Millard Co.: north of Fossil Mountain | 38.88 | -113.47 | 8 | 11,816,706  (2×100) |
| *R. melanophthalma* | mela_REF | USA, Colorado, Rocky Mountain NP, Larimer County, Estes Park, Old Fall River Rd. | 40.44 | -105.74 | ‘R’ | 17,662,538  (2×250) |
| *R. novomexicana* | novo_8684d | USA, New Mexico, Taos Co.: Wheeler Peak Wilderness Area, east side | 36.65 | -105.38 | 12 | 8,867,510  (2×250) |
| *R. occulta* | occu_8807_1 | USA, Nevada, White Pine Co.: Humboldt-Toiyabe National Forest, vic. of Cave Mountain Repeater station | 39.17 | -114.61 | 7 | 11,909,676  (2×100) |
| *R. occulta* | occu_8807_4 | USA, Nevada, White Pine Co.: Humboldt-Toiyabe National Forest, vic. of Cave Mountain Repeater station | 39.17 | -114.61 | 7 | 8,701,458  (2×100) |
| **TAXON** | **DNA_CODE** | **LOCALITY** | **LAT.** | **LON.** | **SITE (Fig. S1)** | **# of filtered reads** |
| *R. parilis* | pari_8665n | USA, Nevada, Nye Co.: Humboldt-Toiyabe National Forest, Table Mountain Wilderness Area, vicinity of Barley Creek | 38.63 | -116.67 | 6 | 9,172,938  (2×100) |
| *R. parilis* | pari_8803 | USA, Utah, Utah Co.: Wasatch-Cache National Forest, American Fork Canyon | 40.43 | -111.73 | 3 | 11,338,144  (2×100) |
| *R. parilis* | pari_8805 | USA, Utah, Utah Co., Wasatch-Cache National Forest, Lone Peak Wilderness Area, vic. of Box Elder Peak | 40.48 | -111.71 | 2 | 12,418,316  (2×100) |
| *R. parilis* | pari_8806 | USA, Utah, Utah Co., Wasatch-Cache National Forest, Lone Peak Wilderness Area, vic. of Box Elder Peak | 40.48 | -111.71 | 2 | 8,896,670  (2×100) |
| *R. polymorpha* | poly_8663o | USA, Utah, Duchesne Co.: Ashley National Forest, South Unit, along Nutter's Ridge Road | 40 | -110.42 | 4 | 14,563,134  (2×100) |
| *R. polymorpha* | poly_8663t | USA, Utah, Duchesne Co.: Ashley National Forest, South Unit, along Nutter's Ridge Road | 40 | -110.42 | 4 | 18,990,876  (2×100) |
| *R. polymorpha* | poly_8665u | USA, Nevada, Nye Co.: Humboldt-Toiyabe National Forest, Table Mountain Wilderness Area, vic. of Barley Creek | 38.63 | -116.67 | 6 | 10,592,040  (2×100) |
| *R. polymorpha* | poly_8668g | USA, Nevada, Nye Co.: Humboldt-Toiyabe N.F., Table Mountain Wilderness Area, vic. of Mosquito Creek | 38.81 | -116.68 | 5 | 20,623,366  (2×100) |
| *R. polymorpha* | poly_8807_3 | USA, Nevada, White Pine Co.: Humboldt-Toiyabe National Forest, vic. of Cave Mountain Repeater station | 39.17 | -114.61 | 7 | 7,330,722  (2×100) |
| *R. polymorpha* | poly_8935b | USA, Idaho, Lemhi Valley, vic. of Birch Creek Campgroup | 44.16 | -112.92 | 1 | 6,976,832  (2×100) |
| **TAXON** | **DNA_CODE** | **LOCALITY** | **LAT.** | **LON.** | **SITE (Fig. S1)** | **# of filtered reads** |
| *R. porteri* | port_8663a | USA, Utah, Duchesne Co.: Ashley National Forest, South Unit, along Nutter's Ridge Road | 40.001 | -110.42 | 4 | 13,560,054  (2×100) |
| *R. porteri* | port_8668a | USA, Nevada, Nye Co.: Humboldt-Toiyabe N.F., Table Mountain Wilderness Area, vic. of Mosquito Creek | 38.81 | -116.68 | 5 | 12,551,072  (2×100) |
| *R. porteri* | port_8795 | USA, Utah, Sevier Co.: north of Fish Lake National Forest Boundary along US Route 72 | 38.7 | -111.41 | 9 | 9,924,428  (2×100) |
| *R. porteri* | port_8796 | USA, Utah, Sevier Co.: north of Fish Lake National Forest Boundary along US Route 72 | 38.7 | -111.41 | 9 | 9,994,784  (2×100) |
| *R. porteri* | port_8797 | USA, Utah, Sevier Co.: north of Fish Lake National Forest Boundary along US Route 72 | 38.7 | -111.41 | 9 | 12,529,538  (2×100) |
| *R. shushanii* | shush_8664_2 | USA, Utah, Wayne Co.: Thousand Lakes Mountain, at summit | 38.41 | -111.48 | 11 | 9,812,538  (2×100) |
| *R. shushanii* | shush_8664_3 | USA, Utah, Wayne Co.: Thousand Lakes Mountain, at summit | 38.41 | -111.48 | 11 | 12,016,870  (2×100) |
| *R. shushanii* | shush_8664_4 | USA, Utah, Wayne Co.: Thousand Lakes Mountain, at summit | 38.41 | -111.48 | 11 | 14,964,922  (2×100) |
| *R. shushanii* | shush_8664_5 | USA, Utah, Wayne Co.: Thousand Lakes Mountain, at summit | 38.41 | -111.48 | 11 | 8,284,106  (2×100) |
| *R. shushanii* | shush_8664_6 | USA, Utah, Wayne Co.: Thousand Lakes Mountain, at summit | 38.41 | -111.48 | 11 | 10,780,442  (2×100) |
| ***Protoparmeliopsis peltata* (outgroup)** | pelt_9101 | USA, Utah, Tooele Co.: Wasatch-Cache National Forest, east of Deseret Peak Wilderness Area, along South Willow Creek Canyon Road | 40.51 | -112.55 | NA | 19,247,366  (2×250) |
| ***R. subdiscrepans***  **(outgroup)** | subd_9052 | USA, Nevada, Humboldt Co.: Humboldt-Toiyabe National Forest, Santa-Rosa/Paradise Peak Wilderness Area, along Rebel Creek | 41.61 | -117.75 | NA | 21,545,594  (2×250) |

**Table S2.** Percent of the *Rhizoplaca melanophthalma* (specimen = ‘mela_REF’) reference genome assembly covered in the RealPhy assembly. The reference genome was comprised only of contigs > 5,000 base pairs and representing over 97% of core eukaryotic genes (including partials).

| Taxon | Specimen ID | genome coverage (proportion) | # of filtered PE reads |
| --- | --- | --- | --- |
| *R. haydenii* | hay_8678 | 0.697 | 10,226,038 (2×100) |
| *R. haydenii* | hay8935p | 0.668 | 8,549,740 (2×100) |
| *R. melanophthalma* | mel_8668b | 0.610 | 9,501,798 (2×100) |
| *R. melanophthalma* | mel_8800 | 0.692 | 6,211,434 (2×100) |
| *R. melanophthalma* | mel_8801 | 0.917 | 12,371,904 (2×100) |
| *R. melanophthalma* | mel_8802 | 0.848 | 11,653,316 (2×100) |
| *R. melanophthalma* | mel_8810 | 0.753 | 11,816,706 (2×100) |
| *R. melanophthalma* | mela_REF | 0.999 | 17,662,538 (2×250) |
| *R. novomexicana* | nov_8684d | 0.688 | 8,867,510 (2×250) |
| *R. occulta* | occ_8807_1 | 0.688 | 11,909,676 (2×100) |
| *R. occulta* | occ_8807_4 | 0.623 | 8,701,458 (2×100) |
| *R. parilis* | par_8665n | 0.616 | 9,172,938 (2×100) |
| *R. parilis* | par_8803 | 0.683 | 11,338,144 (2×100) |
| *R. parilis* | par_8805 | 0.579 | 12,418,316 (2×100) |
| *R. parilis* | par_8806 | 0.638 | 8,896,670 (2×100) |
| *R. polymorpha* | pol_8663o | 0.727 | 14,563,134 (2×100) |
| *R. polymorpha* | pol_8663t | 0.717 | 18,990,876 (2×100) |
| *R. polymorpha* | pol_8668g | 0.744 | 20,623,366 (2×100) |
| *R. polymorpha* | pol_8807_3 | 0.512 | 7,330,722 (2×100) |
| *R. polymorpha* | pol_8865u | 0.699 | 10,592,040 (2×100) |
| *R. polymorpha* | pol_8935b | 0.618 | 6,976,832 (2×100) |
| *R. porteri* | por_8663a | 0.718 | 13,560,054 (2×100) |
| *R. porteri* | por_8668a | 0.716 | 12,551,072 (2×100) |
| *R. porteri* | por_8795 | 0.691 | 9,924,428 (2×100) |
| *R. porteri* | por_8796 | 0.686 | 9,994,784 (2×100) |
| *R. porteri* | por_8797 | 0.708 | 12,529,538 (2×100) |
| *R. shushanii* | shu_8664_2 | 0.728 | 9,812,538 (2×100) |
| *R. shushanii* | shu_8664_3 | 0.738 | 12,016,870 (2×100) |
| *R. shushanii* | shu_8664_4 | 0.766 | 14,964,922 (2×100) |
| *R. shushanii* | shu_8664_5 | 0.738 | 8,284,106 (2×100) |
| *R. shushanii* | shu_8664_6 | 0.741 | 10,780,442 (2×100) |
| ***Protoparmeliopsis peltata* (outgroup)** | pelt_9101 | 0.047 | 19,247,366 (2×250) |
| ***R. subdiscrepans* (outgroup)** | subd_9052 | 0.186 | 21,545,594 (2×250) |

**Supplementary text:**

**Supplementary text: 303 core eukaryotic genes extracted from ‘CEGMA’ dataset**

KOG0002.10, KOG0018.2, KOG0019.7, KOG0047.11, KOG0073.6, KOG0077.7, KOG0084.2, KOG0092.5, KOG0094.4, KOG0102.3, KOG0122.7, KOG0142.7, KOG0173.2, KOG0174.1, KOG0175.8, KOG0177.13, KOG0179.7, KOG0180.6, KOG0183.5, KOG0184.8, KOG0185.16, KOG0188.9, KOG0190.1, KOG0225.12, KOG0261.15, KOG0264.2, KOG0271.4, KOG0279.10, KOG0285.11, KOG0289.20, KOG0292.5, KOG0302.9, KOG0313.4, KOG0328.6, KOG0329.5, KOG0331.5, KOG0344.1, KOG0357.5, KOG0358.2, KOG0361.3, KOG0362.4, KOG0364.1, KOG0365.4, KOG0366.10, KOG0367.12, KOG0371.2, KOG0373.3, KOG0376.3, KOG0394.5, KOG0397.10, KOG0400.16, KOG0402.12, KOG0419.5, KOG0420.10, KOG0424.1, KOG0441.9, KOG0450.4, KOG0460.1, KOG0466.5, KOG0469.7, KOG0477.3, KOG0481.1, KOG0495.4, KOG0523.19, KOG0524.8, KOG0534.2, KOG0544.11, KOG0556.2, KOG0563.9, KOG0567.18, KOG0622.17, KOG0625.1, KOG0631.5, KOG0650.3, KOG0659.7, KOG0675.10, KOG0683.2, KOG0728.1, KOG0729.4, KOG0734.4, KOG0756.6, KOG0758.6, KOG0767.5, KOG0780.6, KOG0784.8, KOG0785.6, KOG0787.15, KOG0788.4, KOG0815.12, KOG0829.17, KOG0853.8, KOG0857.8, KOG0861.3, KOG0876.6, KOG0878.22, KOG0880.2, KOG0898.10, KOG0922.1, KOG0927.5, KOG0935.3, KOG0937.11, KOG0938.6, KOG0948.8, KOG0959.5, KOG0969.2, KOG0985.10, KOG0989.5, KOG0996.10, KOG1036.17, KOG1047.5, KOG1058.11, KOG1062.6, KOG1088.17, KOG1099.19, KOG1112.7, KOG1123.1, KOG1131.4, KOG1149.12, KOG1158.4, KOG1159.1, KOG1180.4, KOG1235.5, KOG1255.8, KOG1268.15, KOG1291.8, KOG1299.3, KOG1300.7, KOG1335.9, KOG1342.1, KOG1349.11, KOG1350.9, KOG1351.5, KOG1353.6, KOG1358.5, KOG1370.8, KOG1374.4, KOG1390.5, KOG1394.7, KOG1430.13, KOG1433.6, KOG1439.4, KOG1448.11, KOG1458.7, KOG1463.5, KOG1468.12, KOG1487.3, KOG1498.6, KOG1506.14, KOG1523.10, KOG1526.9, KOG1531.14, KOG1532.1, KOG1534.5, KOG1535.2, KOG1540.6, KOG1541.14, KOG1549.3, KOG1555.2, KOG1556.1, KOG1562.17, KOG1567.9, KOG1597.2, KOG1637.12, KOG1641.11, KOG1643.16, KOG1644.13, KOG1647.10, KOG1654.4, KOG1662.1, KOG1664.5, KOG1678.6, KOG1723.11, KOG1727.2, KOG1733.3, KOG1746.13, KOG1753.14, KOG1754.14, KOG1755.3, KOG1758.8, KOG1760.6, KOG1762.1, KOG1769.6, KOG1770.16, KOG1774.6, KOG1779.18, KOG1780.3, KOG1782.7, KOG1784.12, KOG1872.4, KOG1889.5, KOG1936.12, KOG1980.3, KOG2014.6, KOG2035.8, KOG2047.17, KOG2067.9, KOG2276.13, KOG2292.7, KOG2303.5, KOG2309.13, KOG2321.6, KOG2446.2, KOG2451.5, KOG2467.11, KOG2472.5, KOG2519.8, KOG2531.7, KOG2537.6, KOG2572.7, KOG2574.7, KOG2575.1, KOG2613.13, KOG2623.10, KOG2636.12, KOG2638.6, KOG2680.2, KOG2700.1, KOG2707.19, KOG2711.3, KOG2719.6, KOG2732.20, KOG2738.9, KOG2754.6, KOG2757.4, KOG2767.4, KOG2772.9, KOG2775.7, KOG2784.10, KOG2785.8, KOG2792.18, KOG2803.6, KOG2807.16, KOG2825.16, KOG2833.3, KOG2874.1, KOG2877.1, KOG2906.2, KOG2908.9, KOG2909.3, KOG2916.3, KOG2930.7, KOG2952.12, KOG2957.12, KOG2967.13, KOG2971.1, KOG2981.6, KOG2988.13, KOG3013.2, KOG3022.14, KOG3031.5, KOG3049.2, KOG3064.2, KOG3079.5, KOG3157.10, KOG3180.2, KOG3185.6, KOG3188.4, KOG3189.9, KOG3205.15, KOG3218.17, KOG3222.11, KOG3229.5, KOG3232.3, KOG3237.3, KOG3239.10, KOG3271.4, KOG3273.14, KOG3275.13, KOG3283.10, KOG3284.6, KOG3291.13, KOG3295.5, KOG3301.2, KOG3311.11, KOG3318.8, KOG3330.3, KOG3343.4, KOG3349.9, KOG3387.10, KOG3400.12, KOG3404.9, KOG3405.6, KOG3406.1, KOG3418.1, KOG3428.11, KOG3430.6, KOG3432.6, KOG3436.7, KOG3442.3, KOG3449.11, KOG3453.6, KOG3457.9, KOG3459.6, KOG3463.16, KOG3475.7, KOG3479.2, KOG3489.5, KOG3497.16, KOG3498.10, KOG3499.17, KOG3503.23, KOG3954.6, KOG4392.19

**Supplementary text: complete materials and methods**

*Culture Isolation for reference genome*

For this study, an axenic culture representing the mycobiont taxon *R. melanophthalma* s. str. (‘mela_REF’) was used to provide reference genomic data: sample No. 074S.5, inoculum No. RHIZMEL-1, culture collection number (cryostock) LMCC0506, (first) DNA extraction number L1056; origin data: U.S.A., Colorado, Rocky Mountain National Park, Larimer County, Estes Park, Old Fall River Rd. Saxicolous on boulders right above the treeline, 3494 m a.s.l. (N 40.4407, W 105.7465), collected by Fernando Fernandez Mendoza, 18 July 2009. The lichen thallus was carefully inspected for lichenicolous fungal infections and a single areola was removed with a sterile razor blade and put into an Eppendorf tube. The isolation protocol followed the ‘thallus fragments’ method as in ^1^. In short, the pieces, about 2 mm^2^, were washed three times for 15 minutes in distilled sterile water on a shaking bath, followed by a 30 minutes washing step with 500 µl of 1:10 dilution of Tween 80 to remove the possible external contaminations of bacteria and yeast ^2^. A final washing step was carried out twice in distilled sterile water for 15 minutes. Clean fragments were homogenized in water (ca. 2-3 ml) using a mortar and pestle. The suspension containing small pieces of the cortex, the medulla and the algal layer was filtered through two sieves of 500 and 150 m mash size, respectively. Single fragments of the mycobiont hyphae of about 150 μm in size were picked up with bamboo sticks under a dissecting microscope and were individually inoculated on slanted agar in test tubes. In order to promote the growth of the mycobiont the dissected fragments were inoculated on three x different media: *Trebouxia* Medium (TM, ^3^, Malt Yeast Extract Medium (MY, ^3^ and Lilly and Barnett´s Medium (LBM, ^4^. Four tubes of the same medium were inoculated, resulting in a total of 24 tubes (inocula) for the original sample. The tubes were incubated in a growing chamber at 20 °C, with a light-dark regime of 14:10 hours with light intensity of 60-100 µmol photons m^-2^s^-1^ and 60% humidity. After five to eight months, the inocula reached about 1-3 mm in diameter, and the *R. melanophthalma* s. str. culture was sub-cultured on multiple plates In order to obtain a sufficient amount of DNA to be used for genomic studies. The subcultures were set on agar plates using the same growth medium where the inoculum has grown successfully, with each plate containing three inocula of the same original culture/isolate. The cultured strains are deposited at the University of Graz in the culture collection of the author LM (LMCC) and are preserved both as fresh cultures and as cryostocks.

*Taxonomic sampling*

A total of 30 specimens representing eight of the nine described species within the *R. melanophthalma* species complex, including a single representative of *R. novomexicana*, were collected from sites throughout western North America (Table S1 online; Fig. S4 online). We were unable to obtain fresh material representing the vagrant taxon *Rhizoplaca idahoensis*, which has previously been shown to be closely related to *R. haydenii* ^5^. Two additional *Rhizoplaca* s. lat. species were included as outgroups, *Protoparmeliopsis peltata* (Ramond) Arup, Zhao Xin & Lumbsch, and *R. subdiscrepans* (Nyl.) R. Sant*. Rhizoplaca novomexicana* was shown to be the earliest diverging lineage in the *Rhizoplaca melanophthalma* species group (see Results), and was used to root topologies of the focal group.

*DNA extraction and sequencing*

DNA isolation from the axenic *R. melanophthalma* s. str. culture followed the CTAB protocol ^6^. Total genomic DNA was obtained from all field-collected samples using the Prepease DNA Isolation Kit (USB, Cleveland, Ohio, USA) following the leaf extraction protocol. The identity of each DNA extraction, including the reference cultured mycobionts, was tested by sequencing the nuclear ITS rDNA region using the primers ITS1f ^7^ and ITS4 ^8^. Genomic libraries for the *R. melanophthalma* s. str. reference culture, *R. novomexicana*, *R. subdiscrepans*, and *P. peltata* were prepared using Illumina’s TruSeq DNA LT Sample Prep Kit and following the manufacture’s instructions for 250-bp paired-end (PE) reads from a 700-bp insert size. Libraries were sequenced on Illumina’s MiSeq platform using the Illumina’s MiSeq v2 Reagent Kit at the Pritzker Laboratory for Molecular Systematics at the Field Museum (Chicago, IL, USA). Library preparation and sequencing of genomic DNA from the remaining 30 samples was completed at the Georgia Genomics Facility (http://dna.uga.edu/). Libraries were constructed using an in-house method and libraries were pooled and sequenced on a single lane of Illumina HiSeq2000, 100-bp paired-end reads with a 350-bp insert size.

*Read filtering and genome assemblies*

All PE reads were filtered using TRIMMOMATIC v0.33 ^9^ before assembly to remove low quality reads and/or included contamination from Illumina adaptors using the parameters recommended by the authors. The genome size of *R. melalnophthalma* s. str. was estimated from filtered PE reads using the Perl script “estimate_genome_size.pl” (https://github.com/josephryan/estimate_genome_size.pl).

A reference draft genome was assembled using PE Illumina reads from the axenic *R. melanophthalma* s. str. culture (‘mela_REF’) using the RAY v2.3.1 assembler ^10,11^ with a kmer value of 41 and the remaining parameters set to default values. An exploratory comparison of assemblies using the RAY and SPAdes v3.1.1 ^12^ assemblers, implementing a variety of kmer values, indicated that the selected RAY assembly was most complete in terms of core eukaryotic genes (CEG; ^13^ and closest to the estimated genome size.

*Phylogenomic data matrices*

Three phylogenomic datasets were assembled for this study: ‘RealPhy’; ‘CEGMA’; and ‘100, 1kb’ (Fig. 1).

The most comprehensive nuclear phylogenomic dataset – ‘RealPhy’ – was constructed using the program RealPhy v1.12 ^14^. After excluding the contig containing the mitochondrial genome, all contigs from the reference (‘mela_REF’) genome assembly larger than five kb were used as the reference. PE reads from all the remaining specimens were mapped to the reference using Bowtie 2.1.0 ^15^, implementing the following parameters in RealPhy v1.12, implementing Bowtie 2.1.0 for read mapping and the following parameters: -readLength 75 –perBaseCov 5 –gapThreshold 0.2, with the remaining parameters set to default values.

The ‘CEGMA’ genomic data matrix was constructed using the Core Eukaryotic Gene Mapping Approach (CEGMA; ^13,16^. Proteins included in CEGMA represent 458 eukaryotic orthologous groups (KOGs) that are conserved among eukaryotes and provide a potentially informative phylogenomic markers ^17^. Reads from each library were mapped to CEGMA regions on the reference assembly using Bowtie 2.1.0 ^15^. Consensus sequences for each CEG region, including introns and adjoining regions, were generated using the bam2consensus tool from the bambam package ^18^ and aligned using the program MUSCLE ^19^. The ‘CEGMA’ matrix was concatenated from a total of 430 CEG alignments with the program FASconCAT ^20^. For individual CEG open reading frame (ORF) extraction, exons were extracted from individual CEG alignments and concatenated. Inserts in all sequences, other than the reference, were removed. All alignments were eliminated that that did not start with ATG; end with TGA, TAA, or TAG; contained more than one stop codon in translation; or were not divisible by three. A total of 303 CEGs (ORF) alignments remained. A concatenated alignment of all individual 303 CEG ORFs for further phylogenetic analysis were build using FASconCAT.. The complete pipeline is available as a GitHub repository (<https://github.com/felixgrewe/CEGMA_CDS_extract>).

The ‘100,1kb loci’ dataset was assembled from a single 1 Kb genomic region selected from each of the 100 largest contigs from the RAY assembly of the reference genome. Reads from the outgroup taxon *Protoparmeliopsis peltata* were mapped to the ‘mela_REF’ reference genome using the Geneious v8.2 Read Mapper ^21^, with the “medium-low sensitivity/ fast” settings, iterated 5 times. Mappings of the *P. peltata* reads to the 100 largest contigs in the reference genome were examined to identify the first 1 kb regions covered without gaps. Furthermore, each genomic region was evaluated for uniform coverage across the locus and similar coverage among loci to avoid the selection of paralogous or repetitive genomic regions. PE reads from all specimens were mapped to these 100, 1 kb markers from the reference genome using RealPhy v1.12, implementing Bowtie 2.1.0 for read mapping and the following parameters: -readLength 75 –perBaseCov 5 –gapThreshold 0.2, with the remaining parameters set to default values. We assessed phylogenetic informativeness (PI) for each locus in the ‘100,1kb loci’ dataset using the PhyDesign web interface ^22^.

*Phylogenomic inference*

Phylogenetic relationships were inferred using maximum likelihood (ML) and multi-species coalescent species tree approaches. Concatenation can perform as well or better than coalescent-based species tree methods in some cases, particularly in situations where levels of incomplete lineage sorting are low ^23,24^. ML phylogenetic relationships were inferred from the complete the ‘RealPhy’, ‘CEGMA’, and ‘100, 1Kb’ datasets using the program RAxML v8.2.3 ^25^. Individual ML topologies were also inferred for each individual locus in the ‘RealPhy’ and ‘CEGMA’ datasets. For the RAxML analyses of the ‘RealPhy’ and ‘CEGMA’ datasets, we implemented the ‘GTRGAMMA’ model, which includes a parameter for rate heterogeneity, combining 200 separate ML searches to find the optimal tree and evaluating nodal support using 1000 bootstrap pseudo-replicates. We inferred phylogenies from the ‘100,1kb loci’ dataset and the 303 CEGs extracted from the ‘CEGMA’ dataset using partitioned ML analyses in RAxML. We used the program PartitionFinder ^26^ to infer the most appropriate partitioning. We also reconstructed a phylogeny exclusively using third codon positions from the 303 CEGs extracted from the ‘CEGMA’ dataset. RAxML analyses were otherwise performed as described above.

Evolutionary histories of individual genes may differ from the underlying species tree ^27^, and incongruence among individual gene topologies from the ‘CEGMA’ and ‘100, 1kb’ datasets was evaluated using the internode certainty (IC) and relative tree certainty (TCA) metrics from ^28^. The IC value of a given internode reflects its specific degree of incongruence, and the TCA value characterized the global degree of incongruence between trees. Individual gene trees were from the ‘CEGMA’ and ‘100, 1kb’ datasets were estimated using RAxML v8.2.3 ^25^ as described above.

*Species tree inference from phylogenomic data*

Because phylogenetic inferences from concatenated data may disagree with species tree approaches in some cases ^29^, we inferred species-trees for the *R. melanophthalma* group using three approaches based on the multispecies coalescent model, the summary coalescent approaches ASTRAL-II ^30^ and SVDquartets ^31^, along with a Bayesian hierarchical approach, *BEAST ^32^.

We used the summary coalescent model ASTRAL-II v4.7.8 ^30^ to inter a species tree from two sets of unrooted gene trees: (1) gene trees inferred from the alignments of the 430 CEGs (and associated introns and small portions of upstream and downstream regions) identified in this study; and (2) gene trees inferred from each of the ‘100, 1Kb’ loci. ASTRAL-II estimates a species tree given a set of unrooted gene trees and has been shown to be statistically consistent under the multi-species coalescent model ^30^. Individual ML gene trees and bootstrap replicates were inferred using RAxML v8.2.3. We used ASTRAL-II with multi-locus bootstrapping (MLBS) option.

A second summary method, SVDquartets ^31^, infers the quartet trees for all subsets of four species using unlinked multi-locus data, assigning a score to each of the three possible quartet topologies. The quartet topology with the lowest “SVD score” is selected as the true topology for that quartet and the sets of quartet trees are combined into a species tree ^31^. We ran SVDquartets as implemented in PAUP* v4.0a146 using the ‘CEGMA’ and ‘100, 1Kb’ datasets, independently. The SVDquartets + PAUP* analyses implemented the species-tree analysis using taxon partitions option, evaluating 100,000 random quartets, and trees were selected using the QFM quartet amalgamation. Bootstrap support was evaluated with 1000 bootstrap replicates. We also ran SVDquartets as described above on two subsets of the ‘100, 1Kb’ dataset, arbitrarily dividing the 100 loci, into two 50-locus datasets to assess the performance SVDquartets + PAUP* using smaller genomic datasets.

We also estimated species trees using the hierarchical Bayesian model implemented in *BEAST v. 1.8.2 ^32^. *BEAST estimates a species tree directly from the sequence data, incorporating the coalescent process, uncertainty associated with gene trees, and nucleotide substitution model parameters ^32^. *BEAST is computationally intensive, and we arbitrarily divide the ‘100, 1Kb’ dataset, into two 50-locus datasets in order for the analyses to be computationally feasible. The two 50-locus datasets were further divided into four 25-locus datasets to investigate if relatively small phylogenomic datasets – in this case, 25, ca. 1kb genetic markers – provide similar estimates to more comprehensive genomic sampling. Nucleotide substitution models were inferred for each locus using the program PartitionFinder v1.1.1 ^26^ with Akaike Information Criterion model selection. For all *BEAST analyses we selected the birth-death speciation prior, implementing a relaxed lognormal molecular clock. Two independent MCMC analyses were run for a total of 100 million generations, sampling every 1500 steps, and excluding the first 25% of generations from each run as burn-in. We assessed convergence by examining the likelihood plots through time using Tracer v. 1.6 ^33^, and the effective sample sizes (ESS) of parameters. Posterior probabilities (PP) of nodes were computed from sampled trees after burn-in.

*Bayesian species validation*

We estimated the marginal posterior probability of speciation from the individual loci in the ‘100, 1kb’ dataset using the program BP&P v3 ^34,35^. This method accommodates the species phylogeny as well as lineage sorting due to ancestral polymorphism. We used a conservative combination of priors that should favor fewer species by assuming large ancestral population sizes and relatively shallow divergences among species with algorithm 0. The population size parameter (*θ*) was assigned the gamma prior Γ(2, 10) and the divergence time at the root of the species tree (τ_0_) was assigned the gamma prior Γ(2, 2000), while the other divergence time parameters were assigned the Dirichlet prior ^34^. Species trees estimated in SVDquartets + PAUP* and *BEAST analysis were used as the fully resolved guide trees, differing only in the placement of taxa within the closely related ‘*porterii*’ group (see Results). Running the rjMCMC analysis for 50,000 generations with a burn-in of 50,000 produced consistent results across independent analyses initiated with different starting seeds and species trees. Each analysis was run at least twice to confirm consistency between runs. We also ran BP&P as described above on two subsets of the ‘100, 1Kb’ dataset, arbitrarily dividing the 100 loci, into two 50-locus datasets to assess if BP&P provided consistent results across different datasets.

**References**
